# Α 6-month, multicenter, observational study investigating the treatment of venous thromboembolism in Greece (VICTORIA study)

**DOI:** 10.1186/s12959-025-00749-1

**Published:** 2025-06-23

**Authors:** Paraskevi Savvari, Ioannis Skiadas, Evgenia Mavrokefalou, Stavros Kakkos, Ioulia Antoniou, Georgios A. Pitoulias, Effrosyni Dima, Emmanouil Ferdoutsis, Georgios Ntaios, Athanasios Giannoukas, Ourania Kotsiou, Flora Zagouri, Georgios Tsoukalas, Konstantinos Kostikas, Dimitrios Staramos, Haralampos Milionis, Konstantinos Filis, Christos Savopoulos, Ioannis Kakisis, Vasileios Tzilalis, Nikolaos Koulouris, Theophanis Papas, Ioanna Skrapari, Damianos Menegas, The VICTORIA Study Group, Ioannis Kalomenidis, Foteini Malli, Georgios Meletis, Konstantinos Nikolakopoulos, Dimitrios A. Chatzelas, Dimitrios Sagris, Christos Karathanos, Maria Kaparelou, Ilektra Voulgareli, Christos Kyriakopoulos, Panagiotis Theodoridis, Sebastian Filippas Ntekouan

**Affiliations:** 1 Medical Affairs, Pfizer Hellas S.A, 243 Messoghion Ave, Athens, N. Psychiko 154 51 Greece; 2 4th Department of Internal Medicine, Evangelismos Hospital, Athens, Greece; 3 Department of Vascular Surgery, University of Patras Medical School, Patras, Greece; 4 Division of Vascular Surgery, 2nd Surgery Clinic, School of Health Sciences, Faculty of Medicine, Aristotle University of Thessaloniki, Thessaloniki, Greece; 5 1st Department of Critical Care and Pulmonary Services, Evangelismos Hospital, Athens, Greece; 6 Pulmonary Medicine Department, General Hospital of Heraklion “VENIZELEIO”, Heraklion, Greece; 7 Department of Internal Medicine, School of Health Sciences, University of Thessaly, Larissa, Greece; 8 Vascular Surgery Department, Faculty of Medicine, School of Health Sciences, University Hospital of Larissa, University of Thessaly, Larissa, Greece; 9 Respiratory Medicine Department, School of Medicine, University of Thessaly, University Hospital of Larissa, Larissa, Greece; 10 Oncology Department of Clinical Therapeutics, Alexandra Hospital, National Kapodistrian University of Athens School of Medicine, Athens, Greece; 11 4th Respiratory Medicine Department, General Hospital for Chest Diseases of Athens “SOTIRIA”, Athens, Greece; 12 Respiratory Medicine Department, University of Ioannina, Ioannina, Greece; 13 Vascular Surgery Department, “Konstantopouleion” General Hospital of Nea Ionia, Athens, Greece; 14 1st Department of Internal Medicine, School of Medicine, University Hospital of Ioannina, Ioannina, Greece; 15 Vascular Surgery Unit, 1st Department of Propaedeutic Surgery, National and Kapodistrian University of Athens, Hippocration Hospital, Athens, Greece; 16 1st Propaedeutic Internal Medicine Department, AHEPA University General Hospital of Thessaloniki, Thessaloniki, Greece; 17 Vascular Surgery Department, Attikon University Hospital, National and Kapodistrian University of Athens, Athens, Greece; 18 Vascular Surgery Department, 401 General Military Hospital of Athens, Athens, Greece; 19 Respiratory Medicine Department, Rehabilitation Unit, 1st Sotiria Hospital, National and Kapodistrian University of Athens, Athens, Greece; 20 Vascular Surgery Department, “Red Cross” General Hospital of Athens, Athens, Greece; 21 1st Department of Internal Medicine, Evangelismos General Hospital, Athens, Greece

**Keywords:** Anticoagulant, Deep vein thrombosis, Pulmonary embolism, Duration of anticoagulation

## Abstract

**Background:**

Real-world data are needed to inform clinical practice with regards to anticoagulation treatment for persons with venous thromboembolism (VTE).

**Objectives:**

To identify the type and duration of antithrombotic treatment in persons with VTE. Anticoagulation dosage and persistence/adherence were among the secondary objectives.

**Methods:**

A multicenter, observational, prospective study conducted in Greek adults with VTE with two on-site visits -baseline and at three months- and a telephone follow-up at 6 months.

**Results:**

A total of 600 eligible persons were enrolled. The index event was ‘PE only’ in 50%, ‘DVT only’ in 40%, and ‘DVT+PE’ in 10%. Risk factors were categorized as temporary major (21%), temporary minor (37%), and persistent (43%), with active cancer present in 18% of patients. All VTE patients received anticoagulants: 73% received oral anticoagulants (72% DOACs, 1% VKAs) and 70% received parenteral anticoagulants. Treatment was oral only in 30%, parenteral only in 27%, and both in 43%. The most common DOAC was apixaban (47%). Extended anticoagulation (>6 months) was administered to 41% with only 9% (18/198) of those on DOACs receiving a reduced dose. Persistent risk factors predicted extended anticoagulation, while diabetes, COVID-19, and temporary minor risk factors did not. Adherence/persistence rates were similar between DOAC and non-DOAC-treated patients.

**Conclusion:**

VTE was mainly treated with a combination of parenteral and oral anticoagulants. DOACs, primarily apixaban, were the most common oral treatments. Forty percent of patients received extended anticoagulation, mostly at standard dosages. Adherence and persistence rates were high for both DOAC and non-DOAC treatments.

**Supplementary Information:**

The online version contains supplementary material available at 10.1186/s12959-025-00749-1.

## Background

Venous thromboembolism (VTE), which comprises deep vein thrombosis (DVT) and pulmonary embolism (PE), has an incidence of 100–200 per 100.000, with European annual estimates of ~470,000 DVT cases and ~300,000 PE cases [1, 2].

Treatment of acute episodes of VTE typically requires at least a 3-month period of anticoagulation (AC), either with parenteral therapy followed by vitamin K antagonist (VKA) or alternatively direct oral anticoagulant (DOAC) therapy with or without bridging. Continuation of AC treatment beyond the first 6 months (extended-therapy) with a reduced-dose DOAC regimen (i.e. apixaban or rivaroxaban), should be considered for persons with high risk of recurrence e.g. no identifiable risk factor (RF), history of prior VTE episode, or presence of a major persistent pro-thrombotic condition [3–5]. Persons with active cancer have both an increased risk of VTE recurrence and bleeding, comprising a special population for whom extended AC may be considered, provided that periodic assessments of the risk–benefit profile and individual preference are performed [6, 7]. Adherence should be also considered when assessing the recurrence risk, since it is another factor associated with better or worse prognosis [8–12]. Overall, the decision to discontinue or extend AC after the first 3–6 months is case-based, since it depends on the assessment of the recurrence risk over bleeding risk, taking into account preferences and anticipated compliance [4]. Thus, the duration of AC therapy is highly variable into clinical practice [13–16].

The aim of our study was to capture the current practice regarding anticoagulants in VTE. We focused on treatment duration -a yet unanswered question in the guidelines- but also identified types of anticoagulants, dosage, extended treatment predictors and adherence/persistence.

## Methods

### Study design and population

VICTORIA was a non-interventional, epidemiological, primary data collection study. Eligible patients were consented adults who had experienced a VTE (DVT/PE) episode (index event) within 7 days and were managed either as inpatients or outpatients by Internists, Pulmonologists, or Vascular surgeons. The index VTE event was confirmed by a valid diagnostic algorithm and imaging modality as recommended by current European and National guidelines (e.g., compression ultrasonography, computed tomography pulmonary angiogram (CTPA), ascending contrast venography, ventilation perfusion lung scanning, pulmonary angiography). Patients were excluded if they were receiving anticoagulant therapy (due to reasons other than the index, were pregnant or lactating at the time of eligibility assessment.

Data were collected at baseline and at 3 months as well as in a telephone follow-up contact at 6 months nominally.

Each participating site was requested to recruit eligible persons sequentially. Consecutive sampling was employed to reduce selection bias. All assessments were performed as part of routine clinical care and standard clinical practice.

### Objectives and assessments

The primary objective was to capture the type and duration of antithrombotic treatment in VTE patients. Secondary objectives were to describe the dosage of antithrombotic treatment during the standard and extended phase (i.e., ≥ 6 months) and to assess patients’ persistence and adherence to anticoagulants with the Adherence to Refills and Medications Scale (ARMS) [17]. Another secondary objective was to identify predictors of extended treatment and of poor adherence and non-persistence.

The study objectives were assessed in the overall population and the subpopulations of patients with ‘DVT only,’ ‘PE only,’ and concurrent ‘DVT and PE.’ Appropriate dosing schedule (i.e., label-recommended dosage) for the DOACs of interest was defined based on the European summary of product characteristics (SmPC) [18, 19]. The rate of extended AC beyond 6 months was defined as the proportion of patients continuing their AC at the 6-month study visit. Poor adherence was defined as ARMS-7 score > 7. Participants’ persistence to antithrombotic medication was defined as renewal of the antithrombotic prescription before the end of drug supply and was assessed both by National electronic prescription records and participants’ self-report.

### Statistical methods

Since this was a descriptive study, no formal statistical hypothesis testing was applied. A sample of 600 patients was considered adequate for addressing study objectives, with a margin of error not exceeding 4% at 95% confidence level based on the normal approximation.

Continuous variables are presented using descriptive statistics [mean (standard deviation, SD) and median (interquartile range, IQR) for normally and non-normally distributed data, respectively] and categorical variables are displayed as frequencies. For non-normally distributed continuous variables (based on Shapiro–Wilk test), a uniform presentation of median (IQR) was applied. Median time on DOAC treatment was estimated using the Kaplan–Meier method.

The association of factors of interest with extended anticoagulant and poor adherence and/or non-persistence to AC was examined using logistic regression analyses. For multivariable analyses, a stepwise procedure was utilized to derive the best fitted model based on Akaike information criterion (AIC). The variables for the initial step were selected based on the data missing rate < 5%, and absence of collinearity among variables.

All statistical tests were two-sided and performed at a 0.05 significance level. All statistical analyses were performed using SAS v9.4 (SAS Institute, Cary, NC).

## Results

### Disposition and care setting

**Fig. 1:**
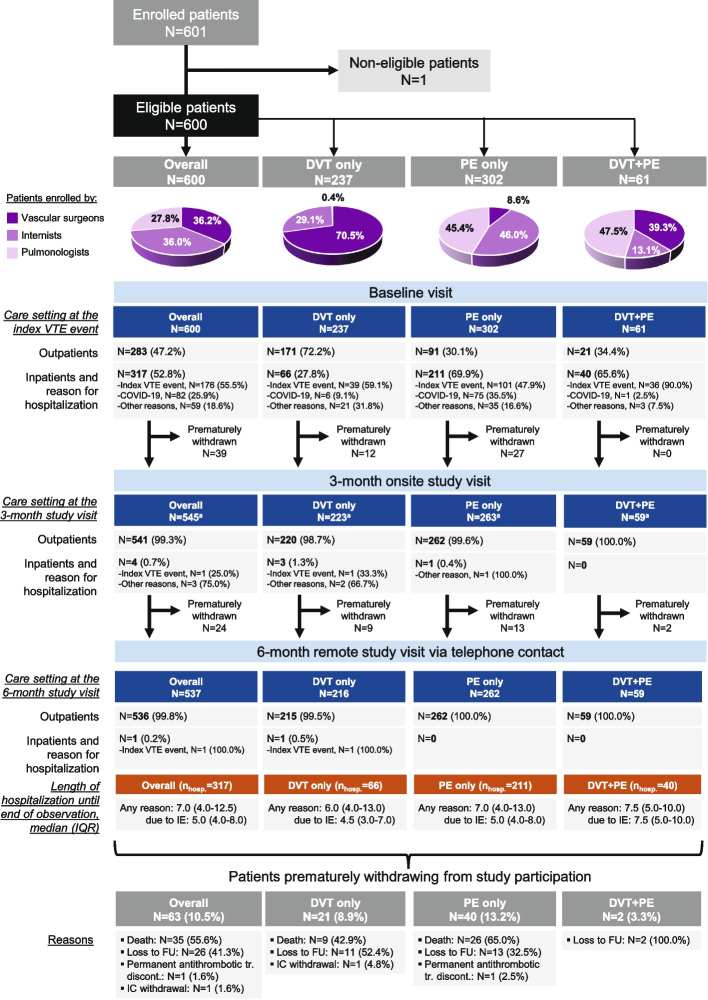
Patient disposition in the study and care setting. ^a^16 patients (DVT only: 2; PE only: 12; DVT + PE: 2) did not attend the 3-month visit but continued study participation. Abbreviations: DVT, Deep Vein Thrombosis; FU, Follow-Up; IC, Informed Consent; IE, Index (VTE) Event; IQR, Interquartile Range; N, number of patients with available data; nhosp., number of hospitalized patients; PE, Pulmonary Embolism; tr., treatment; VTE, Venous Thromboembolism

A total of 601 participants were enrolled by 20 public departments from 19 June 2020 to 23 March 2022. All but one of the participants (*N* = 600) were eligible comprising the full analysis set. Internists, Pulmonologists and Vascular surgeons, enrolled 36.0% (*n* = 216), 27.8% (*n* = 167) and 36.2% (*n* = 217) of eligible patients, respectively. The index was ‘PE only’ in 50.3% (*n* = 302), ‘DVT only’ in 39.5% (*n* = 237), and ‘both DVT and PE’ in 10.2% (*n* = 61) of the participants (Fig. 1). The median (IQR) time from index diagnosis confirmation to baseline visit was 2.0 (0.0–4.0) days for the overall population, and 0.0 (0.0–3.0), 3.0 (1.0–5.0) and 2.0 (0.0–3.0) days for the DVT only, PE only and DVT + PE subpopulations, respectively. The median (IQR) time from index diagnosis to 3- and 6-month follow-up visits was 3.1 (3.0–3.2) and 6.1 (6.0–6.2) months, respectively.

At index diagnosis 47.2% (*n* = 283) of the participants were treated at the out-hospital setting and 52.8% (*n* = 317) at the in-hospital setting; among the latter, the primary reason for hospitalization was the index for 55.5% (176/317), COVID-19 infection for 25.9% (82/317), and other reasons for 18.6% (59/317) (Fig. 1). All but one of the hospitalized participants had been discharged by the end of study observation period after a median (range) length of hospital stay (LOS) of 7.0 (1.0–101.0) days. Median LOS due to index was 5.0 days (range: 1.0–42.0), whereas the respective LOS for reasons other than index was 11.5 days (range: 1.0–101.0; IQR: 6.0–17.0) (Fig. 1).

A total of 10.5% (63/600) of participants were censored prematurely withdrawn from the study after a median (IQR) of 15.0 (6.0–36.0) days since the baseline visit with the most frequent reasons being death (55.6%; 35/63) and loss of follow-up (41.3%; 26/63) (Fig. 1).

Participants disposition, details on the care setting across the study visits and reasons of premature withdrawal, are provided in Fig. 1.

### Participant characteristics and RFs

Of the overall population, 56.3% were males, 30.2% were aged > 75 years and 29.0% had body mass index ≥ 30 kg/m^2^(obese). Participant characteristics are summarized overall and by type of index in Table 1. Small differences were noted between subgroups, in a purely descriptive manner, since statistical significance was not assessed. Specifically, the following characteristics were observed at a higher frequency in ‘PE ± DVT’ patients than ‘DVT only’ participants: male gender (60.1% versus 50.6%), pulse rate ≥ 100 beats/min (19.9% versus 3.3%), arterial oxyhaemoglobin saturation < 90% (9.3% versus 0.5%), and haemodynamic instability (11.8% versus 1.3%). On the other hand, creatinine clearance < 60 mL/min was observed at a higher frequency in the ‘DVT only’ subgroup (30.8% versus 20.2% in the ‘PE ± DVT’ subgroup).

**Table 1:** Patient and disease characteristics in the overall population and by type of index VTE event

Variable	Overall (*N* = 600)	DVT only (*N* = 237)	PE only (*N* = 302)	DVT & PE (*N* = 61)
Demographic characteristics at index VTE event diagnosis				
Male sex, n/N (%)	338/600 (56.3)	120/237 (50.6)	182/302 (60.3)	36/61 (59.0)
Age, median (IQR), years	65.1 (52.9–77.3)	64.4 (53.2–77.0)	66.5 (54.0–78.3)	61.7 (47.2–68.7)
Age > 75 years, n/N (%)	181/600 (30.2)	71/237 (30)	101/302 (33.4)	9/61 (14.8)
BMI (kg/m^2^), median (IQR)	27.7 (25.0–31.2)	27.9 (25.3–31.1)	27.5 (24.9–30.9)	27.7 (24.8–32.8)
BMI ≥ 30 (kg/m2), n/N (%)	174/600 (29.0)	69/237 (29.1)	83/302 (27.5)	22/61 (36.1)
Vital signs at index VTE event diagnosis				
SBP (mmHg), median (IQR)	128.0 (120.0–140.0)	130.0 (120.0–142.0)	125.0 (111.0–140.0)	129.5 (120.0–136.5)
SBP < 100 mmHg, n/N (%)	16/564^b^ (2.8)	3/214 (1.4)	13/290 (4.5)	
DBP (mmHg), median (IQR)	78.0 (70.0–84.5)	80.0 (70.0–85.0)	75.0 (70.0–80.0)	80.0 (70.0–85.0)
PR (beats/min), median (IQR)	82.0 (73.0–91.0)	79.0 (70.0–86.0)	85.0 (75.0–96.0)	85.0 (73.0–94.0)
PR ≥ 100 beats/min, n/N (%)	76/555^b^ (13.7)	7/209 (3.3)	56/285 (19.6)	13/61 (21.3)
RR (breaths/min), median (IQR)	17.0 (14.0–20.0)	17.0 (13.0–18.0)	18.0 (15.0–21.0)	17.0 (13.5–20.0)
SaO2 (%), median (IQR)	96.0 (94.0–98.0)	98.0 (97.0–98.0)	95.0 (92.0–97.0)	96.0 (94.0–97.0)
SaO2 < 90%, n/N (%)	33/535^b^ (6.2)	1/191 (0.5)	29/284 (10.2)	3/60 (5.0)
Laboratory parameters at index VTE event diagnosis				
Hb (g/dL), median (IQR)	12.6 (11.2–14.2)	12.5 (11.2–13.9)	12.8 (11.2–14.1)	12.5 (11.5–14.6)
D-dimer (μg/mL), median (IQR)	3.6 (1.6–7.1)	3.4 (1.6–6.6)	3.5 (1.5–7.0)	4.8 (3.3–9.2)
D-dimer ≥ 0.5 μg/mL, n/N (%)	332/348^b^ (95.4)	97/99 (98.0)	194/207 (93.7)	41/42 (97.6)
PLT (10^3^/μL), median (IQR)	247.0 (197.0–310.0)	246.0 (195.0–290.0)	251.0 (198.0–315.5)	236.0 (190.0–310.0)
P-Cr (mg/dL), median (IQR)	0.9 (0.7–1.1)	0.9 (0.7–1.1)	0.9 (0.7–1.1)	0.9 (0.7–1.1)
CrCl^a^ (mL/min), median (IQR)	88.0 (65.5–112.0)	80.3 (55.0–120.0)	89.0 (69.0–112.0)	89.0 (71.0–112.0)
CrCl < 60 mL/min, n/N (%)	58/260^b^ (22.3)	16/52 (30.8)	36/175 (20.6)	6/33 (18.2)
Characteristics of the index VTE event at initial presentation, n/N (%)				
Symptomatic	528/600 (88.0)	208/237 (87.8)	261/302 (86.4)	59/61 (96.7)
Haemodynamic instability	46/600 (7.7)	3/237 (1.3)	42/302 (13.9)	1/61 (1.6)
Altered mental status	23/600 (3.8)	3/237 (1.3)	16/302 (5.3)	4/61 (6.6)
Location of the index VTE event, n/N (%)				
DVT located in lower limb	256/298 (85.9)	202/237 (85.2)	-	54/61 (88.5)
Proximal DVT (± distal)	171/256 (66.8)	133/202 (65.8)	-	38/54 (70.4)
Unilateral DVT	275/284 (96.8)	218/223 (97.8)	-	57/61 (93.4)
Subsegmental PE location	160/363 (44.1)	-	138/302 (45.7)	22/61 (36.1)

^a^Cockcroft-Gault

^b^Number of patients with available data

*Abbreviations* *BMI* Body Mass Index, *CrCl* Creatinine Clearance, *DBP* Diastolic Blood Pressure, *DVT* Deep Vein Thrombosis, *Hb* Hemoglobin, *IQR* Interquartile Range, *N* number of patients with available data, *n* number of patients with variable, *P-Cr* Plasma Creatinine, *PE* Pulmonary Embolism,* PLT* Platelet count, *PR* Pulse Rate, *RR* Respiratory Rate, *SaO2* Arterial Oxyhaemoglobin Saturation, *SBP* Systolic Blood Pressure, *VTE* Venous Thromboembolism

RFs for VTE were classified as: temporary major, temporary minor, persistent and other based on the European Society of Cardiology (ESC) guidelines [3]. At baseline, 21.0% had ≥ 1 temporary major RF within 3 months before index, 36.5% had ≥ 1 temporary minor RF within 2 months, 43.0% had ≥ 1 persistent RF and 88.3% ≥ 1 other RF. (Table 2). ‘Immobilization within 3 months before the index’ (15.7%), ‘confinement to bed for ≥ 3 days within 2 months before the index’ (14.2%) and ‘cancer’ (22.3%) were among the RFs most commonly reported (> 10% frequency). Among “other risk factors” commonly reported were: ever-smoking (53.5%), arterial hypertension (44.0%), obesity (29.7%), COVID-19 (17.7%), diabetes mellitus (13.7%), and varicose veins (11.0%). History of major bleeding or use of medication predisposing to bleeding was reported in < 10% of participants. Of note, COVID-19 was reported as cause in 17.7% of participants overall (Table 2).

**Table 2:** Comorbidities and risk factors at index VTE event in the overall population

Variable, *n* (%)	Overall (*N* = 600)	DVT only (*N* = 237)	PE only (*N *= 302)	DVT & PE (*N* = 61)
Any identifiable factor that could have contributed to the IE	576 (96.0)	223 (94.1)	292 (96.7)	61 (100.0)
≥ 1 temporary (major or minor) risk factor	278 (46.3)	98 (41.4)	156 (51.7)	24 (39.3)
≥ 1 temporary major risk factor within 3 months before the IE	126 (21.0)	34 (14.3)	81 (26.8)	11 (18.0)
Immobilization^a^	94 (15.7)	19 (8.0)	67 (22.2)	8 (13.1)
Surgery with general anaesthesia for > 30 min	45 (7.5)	14 (5.9)	23 (7.6)	8 (13.1)
Trauma with fractures	22 (3.7)	6 (2.5)	12 (4.0)	4 (6.6)
≥ 1 temporary minor risk factor within 2 months before the IE	219 (36.5)	77 (32.5)	123 (40.7)	19 (31.1)
Bedridden (out of hospital) for ≥ 3 days with an acute illness	85 (14.2)	21 (8.9)	56 (18.5)	8 (13.1)
Prolonged (≥ 6 h) seated immobility	51 (8.5)	20 (8.4)	26 (8.6)	5 (8.2)
Admission to hospital for < 3 days with an acute illness	48 (8.0)	10 (4.2)	34 (11.3)	4 (6.6)
Central venous lines	28 (4.7)	16 (6.8)	12 (4.0)	-
Leg injury^b^ associated with reduced mobility for ≥ 3 days	24 (4.0)	10 (4.2)	11 (3.6)	3 (4.9)
Blood transfusion	21 (3.5)	7 (3.0)	13 (4.3)	1 (1.6)
Erythropoiesis-stimulating agents	17 (2.8)	7 (3.0)	9 (3.0)	1 (1.6)
Superficial vein thrombosis	12 (2.0)	12 (5.1)	-	-
Minor surgery (with general anaesthesia < 30 min)	9 (1.5)	4 (1.7)	4 (1.3)	1 (1.6)
Oestrogen therapy/Contraception (oral contraceptives)	8 (1.3)	6 (2.5)	2 (0.7)	-
≥ 1 persistent risk factor	258 (43.0)	99 (41.8)	132 (43.7)	27 (44.3)
Cancer (past, active, or newly-diagnosed)	134 (22.3)	49 (20.7)	75 (24.8)	10 (16.4)
Cancer (currently active)	106 (17.7)	40 (16.9)	60 (19.9)	6 (9.8)
Chronic pulmonary disease	49 (8.2)	16 (6.8)	31 (10.3)	2 (3.3)
≥ 1 previous VTE episodes in the absence of a major transient or reversible factor	36 (6.0)	12 (5.1)	17 (5.6)	7 (11.5)
Chronic heart failure	28 (4.7)	11 (4.6)	16 (5.3)	1 (1.6)
Active autoimmune disease (other than APS)	22 (3.7)	9 (3.8)	11 (3.6)	2 (3.3)
Known family history of DVT/PE	17 (2.8)	7 (3.0)	8 (2.6)	2 (3.3)
Thrombophilia (other than APS)	10 (1.7)	6 (2.5)	3 (1.0)	1 (1.6)
Inflammatory bowel disease	6 (1.0)	-	2 (0.7)	4 (6.6)
Antiphospholipid syndrome (APS)	2 (0.3)	1 (0.4)	1 (0.3)	-
≥ 1 temporary (major or minor) or persistent risk factor	425 (70.8)	154 (65.0)	229 (75.8)	42 (68.9)
≥ 1 other risk factor^c^	530 (88.3)	206 (86.9)	272 (90.1)	52 (85.2)
Ever-smoking (current and former)	321 (53.5)	124 (52.3)	161 (53.3)	36 (59.0)
Arterial hypertension	264 (44.0)	105 (44.3)	132 (43.7)	27 (44.3)
Obesity (BMI > 30 kg/m^2^)	178 (29.7)	69 (29.1)	87 (28.8)	22 (36.1)
COVID-19	106 (17.7)	9 (3.8)	95 (31.5)	2 (3.3)
Diabetes mellitus	82 (13.7)	31 (13.1)	45 (14.9)	6 (9.8)
Varicose veins	66 (11.0)	40 (16.9)	20 (6.6)	6 (9.8)
Myocardial infarction (within the previous 3 months)	21 (3.5)	9 (3.8)	11 (3.6)	1 (1.6)
History of major bleeding	14 (2.3)	3 (1.3)	10 (3.3)	1 (1.6)
Use of medication(s) predisposing to bleeding at IE presentation	57 (9.5)	19 (8.0)	35 (11.6)	3 (4.9)

^a^Defined as confined to bed in hospital (only bathroom privileges) for ≥ 3 days due to acute illness or acute exacerbation of a chronic illness

^b^without fracture

^c^Reported in > 5 patients total

*Abbreviations* *APS* Antiphospholipid Syndrome, *BMI* Body Mass Index, *DVT* Deep Vein Thrombosis, *IE* Index (VTE) Event, *N* number of patients with available data,* n* number of patients with variable, *PE* Pulmonary Embolism, *VTE* Venous Thromboembolism

In a purely descriptive manner, the prevalence of any temporary RF (major or minor), was higher among ‘PE ± DVT’ patients (49.6%,) than ‘DVT only’ patients (41.4%,) (Table 2). Conversely, no substantial differences between ‘PE ± DVT’ and ‘DVT only’ participants were observed in the frequency of any persistent factor (43.8% versus 41.8%). Of note however, history of prior unprovoked VTE episode(s) was almost double in frequency among participants with both ‘DVT + PE’ (11.5%) compared to ‘DVT only’ and ‘PE only’ participants. With respect to other RFs, COVID-19 infection was reported in 26.7% of ‘PE ± DVT’ participants as opposed to 3.8% in ‘DVT only’ participants. As expected, varicose veins were more common in ‘DVT only’ participants (16.9%) than ‘PE ± DVT’ patients (7.2%) (Table 2).

### VTE treatment strategy

All participants-initiated anticoagulant therapy, while 54.3% (326/600) also received non-pharmacologic interventions, mostly involving compression therapy with stockings/bandages (98.2%; 320/326).

**Fig. 2:**
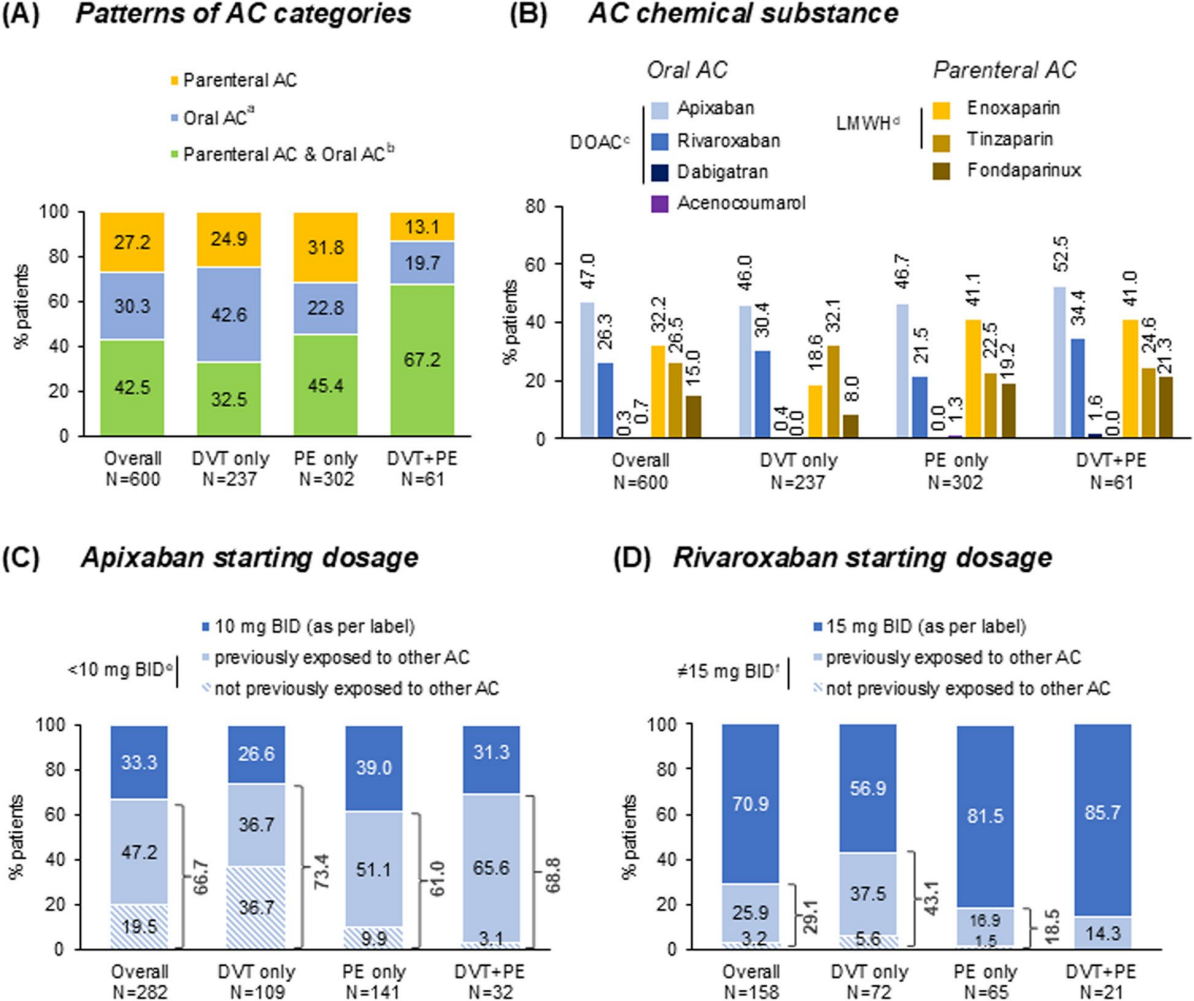
Pharmacologic VTE treatment throughout the study observation period. ^**a**^Two patients (IE: PE only) also received antiplatelet therapy; ^**b**^One patient (IE: PE only) also received systemic thrombolytic therapy; ^**c**^Nine patients (DVT only: 4; PE only: 4; DVT + PE: 1) received ≥ 2 DOACs; ^**d**^Nine patients (DVT only:1; PE only:7; DVT + PE:1) received both LMWH and fondaparinux; either 5 or 2.5 mg BID; f10-20 mg QD and for one patient (IE: PE only) 20 mg BID. Abbreviations: AC, Anticoagulation; BID, twice a day; DOAC, Direct Oral Anticoagulant; DVT, Deep Vein Thrombosis; IE, Index (VTE) Event; LMWH, Low Molecular Weight Heparins; N, number of patients with available data; PE, Pulmonary Embolism; QD, once a day; VTE, Venous Thromboembolism

Anticoagulant treatment patterns included oral anticoagulants only in 30.3% (182/600), parenteral AC only in 27.2% (163/600), and both parenteral and oral anticoagulant in 42.5% (255/600) (Fig. 2A). Among the latter, the median (IQR) time to switch from parenteral to oral anticoagulant was 8.0 (4.0–16.0) days. Most frequently used anticoagulant was DOACs (72.2%; 433/600) and low molecular weight heparin (LMWH) (56.5%; 339/600), followed by fondaparinux (15.0%; 90/600) and VKAs (0.7%; 4/600), with frequencies of active substances shown in Fig. 2B (nine patients received ≥ 2 DOACs). Participants with active cancer were treated with parenteral anticoagulant in 59.4% (63/106) of cases, both parenteral anticoagulant and DOAC in 23.6% (25/106), and DOAC only in 17.0% (18/106).

Parenteral anticoagulant was less common among ‘DVT only’ participants (57,4%; 136/237) compared with ‘PE ± DVT’ (77.7%; 282/363), whereas frequency of oral anticoagulant was similar between the two subgroups [75.1% (178/237) versus 71.3% (259/363)] (Fig. 2A). DOAC utilization rates among ‘DVT only,’ ‘PE only,’ and ‘DVT + PE’ participants were 75.1% (178/237), 66.9% (202/302), and 86.9% (53/61), respectively (Fig. 2B).

Apixaban and rivaroxaban were the most commonly prescribed DOACs (Fig. 2B). Two thirds (66.7%; 188/282) of apixaban-treated participants skipped the lead-in dosing of 10 mg twice daily (BID) [starting with 5 mg BID (62.4%; 176/282) or 2.5 mg BID (4.3%; 12/282)], while a fifth (19.5%; 55/282) skipped the lead-in dosing without having previously received any AC for the management of their index. Almost a third (29.1%; 46/158) of rivaroxaban-treated participants skipped the lead-in dosing of 15 mg BID, while 3.2% (5/158) skipped the lead-in dosing phase without having previously received any other anticoagulant (Fig. 2C-D).

The physician-reported intended duration of anticoagulant at the time of initial treatment decision-making was 3 months for 20.3% of participants and 6 months for 44.5%, while for 34.2% no scheduled stop date was reported (Table 3). The actual median (IQR) anticoagulant duration was 6.0 (5.3–6.2) months. The Kaplan–Meier estimated median time on DOAC treatment was 6.0 [95% confidence interval (CI): 6.0–6.1] months.

**Table 3:** Duration (months) of antithrombotic treatment for the index VTE event

*n* (%)	Overall *(N* = 600)
Scheduled (intended) duration	
3 months	122 (20.3)
6 months	267 (44.5)
9 months	2 (0.3)
12 months	4 (0.7)
No scheduled stop date	205 (34.2)
Actual duration	
< 3 months	52 (8.7)
3–6 months	285 (47.5)
> 6 months	237 (39.5)
Unknown (loss of follow-up)	26 (4.3)

*Abbreviations* *n* number of patients with variable ,*VTE* Venous Thromboembolism

**Fig. 3:**
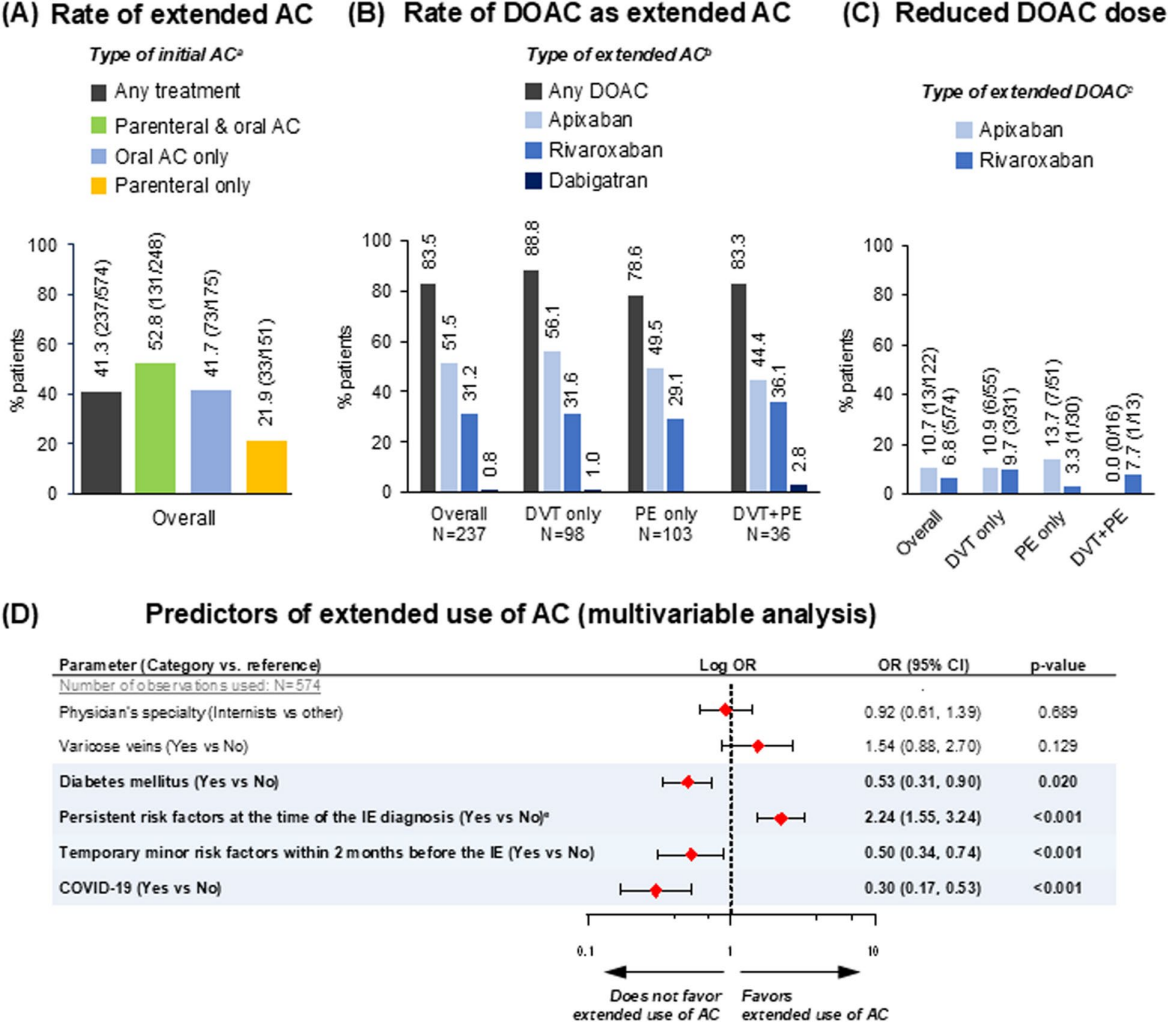
Rate of extended AC beyond 6 months. ^**a**^Denominators represent number of patients receiving the indicated initial AC and with available data on extended AC; ^**b**^Denominators represent number of patients receiving extended AC; ^**c**^Numerators indicate reduced dosing schedule (2.5 mg BID for apixaban and 10 mg QD for rivaroxaban); dabigatran was administered as standard dosing schedule of 150 mg BID in both cases of extended dabigatran (not shown); ^e^Presence of any persistent factor, including active cancer. The modeled probability was use of extended AC beyond months: 'Yes'vs'No’. The following variables were entered in the initial step of the stepwise procedure: Age at IE confirmation (categorical with cut-off 65 years), Gender (Male vs Female), Type of IE (DVT only vs PE ± DVT), Initial presentation of IE (Symptomatic vs asymptomatic), Haemodynamic instability at IE initial presentation (Yes vs No), Temporary major risk factors (Yes vs No), Temporary minor risk factors within 2 months before IE (Yes vs No), Any persistent risk factor (Yes vs No), Diabetes (Yes vs No), Varicose veins (Yes vs No), COVID-19 (Yes vs No), Obesity (Yes vs No), Cigarette smoking (Ever vs Never), Arterial hypertension (Yes vs No). Physician's specialty (Internists vs other) was found to be a confounder in a separate analysis (data not shown); thus; it was forced in the final model (i.e., after the stepwise procedure). Abbreviations: AC, Anticoagulation; AIC, Akaike’s Information Criterion; CI, Confidence Interval; DOAC, Direct Oral Anticoagulant; DVT, Deep Vein Thrombosis; IE, Index (VTE) Event; N, number of patients with available data; OR, Odds Ratio; PE, Pulmonary Embolism

The rate of extended anticoagulant beyond 6 months was 39.5% (237/600) among participants with available data, with respective rates per initial anticoagulant strategy shown in Fig. 3A, and respective rates among evaluable participants in the DVT, PE and DVT + PE subpopulations being 43.4% (98/226), 35.6% (103/289) and 61.0% (36/59). Of the participants treated with extended AC overall, 83.5% (198/237) received DOAC (Fig. 3B), of whom as low as 9.1% (18/198) received a reduced dosing schedule (i.e., apixaban: 2.5 mg BID or rivaroxaban: 10 mg once daily (QD)) (Fig. 3C).

The prevalence of persistent RFs was numerically higher among participants receiving extended anticoagulant (54.9%; 130/237) than those not receiving (34.4%; 116/337). On the contrary, temporary minor RFs were observed more frequently in the latter (43.0%; 145/337) than the former subpopulation (25.7%; 61/237), whereas temporary major RFs were reported in similar frequencies between the extended (18.1%; 43/237) and the non-extended anticoagulant subgroups of participants (22.6%; 76/337). The frequencies of persistent, temporary minor, and temporary major RFs among participants treated with extended reduced-dose DOACs were 50.0% (9/18), 50.0% (9/18), and 11.1% (2/18), respectively, whereas the respective frequencies among those treated with extended standard-dose DOACs were 47.8% (86/180), 21.7% (39/180) and 18.9% (34/180) (data not shown).

Univariable logistic regression analyses of predictors for extended use of anticoagulant are depicted in Figure S1. Multivariable analysis revealed diabetes mellitus, presence of temporary minor RFs, and COVID-19, as negative predictors, and presence of persistent RFs as positive predictor of extended anticoagulant (Fig. 3D). Patients with diabetes, temporary minor RFs, and COVID-19 had 47% (*p* = 0.020), 50% (*p* < 0.001), and 70% (*p* < 0.001) lower odds of receiving extended anticoagulant, whereas the respective odds were more than 2 times higher among participants with persistent RFs (*p* < 0.001) (Fig. 3D).

### Adherence and persistence to antithrombotic medication

**Fig. 4:**
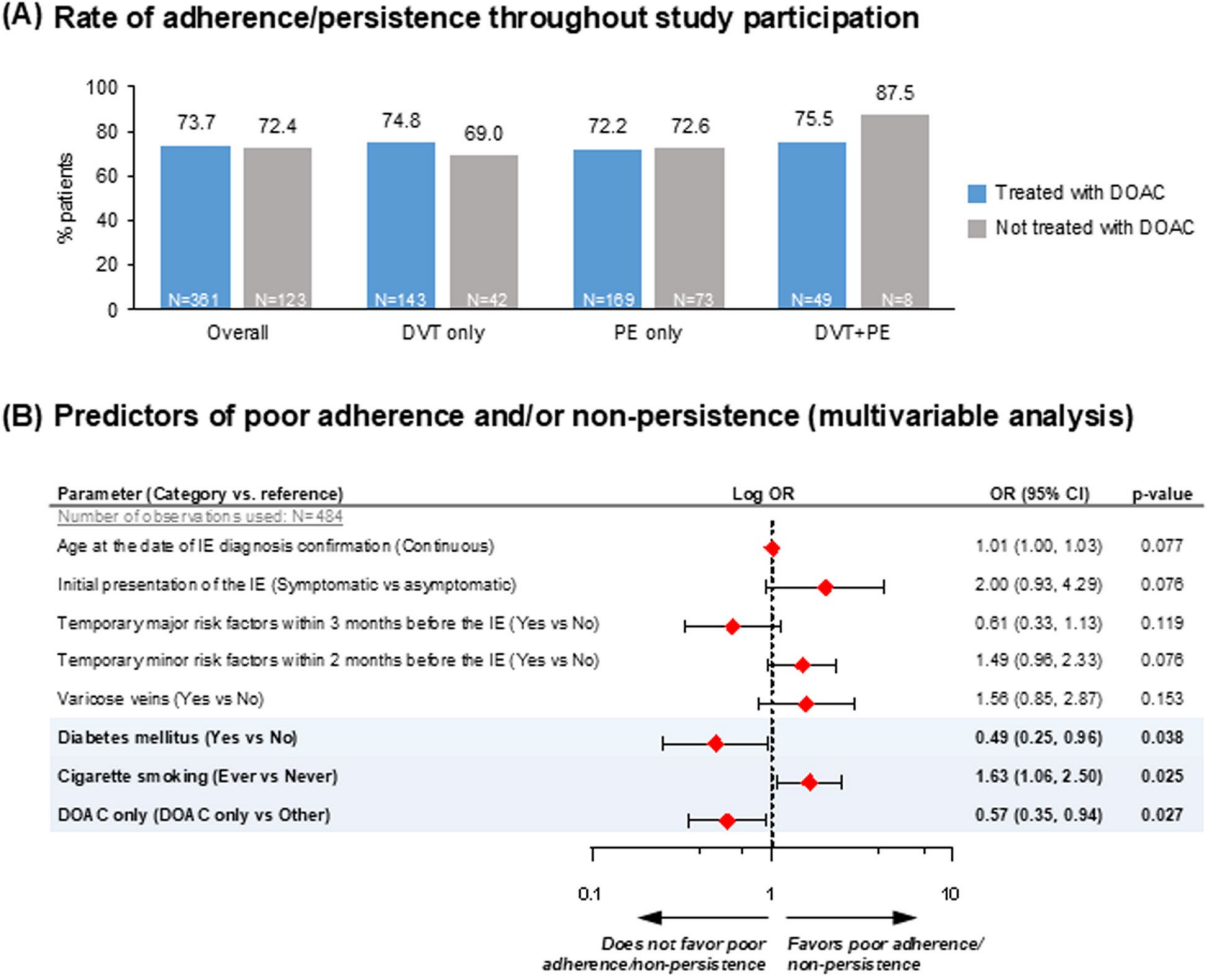
Adherence and persistence to antithrombotic treatment. The modeled probability was poor adherence and/or non-persistence to antithrombotic treatment throughout study participation'yes'versus'no’. The following variables were entered in the initial step of the stepwise procedure: Age at baseline (continuous), Gender (Male vs Female), Type of IE (DVT only vs PE ± DVT), Initial presentation of IE (Symptomatic vs asymptomatic), Temporary major risk factors (Yes vs No), Temporary minor risk factors within 2 months before IE (Yes vs No), Persistent risk factors at the time of IE (Yes vs No), Obesity (Yes vs No), Diabetes (Yes vs No), Varicose veins (Yes vs No), COVID-19 (Yes vs No), Cigarette smoking (Ever vs Never), Obesity (Yes vs No), Cigarette smoking (Ever vs Never), Arterial hypertension (Yes vs No), DOAC only (DOAC only vs Other), Hospitalized at the IE (Yes vs No). Abbreviations: AC, Anticoagulation; CI, Confidence Interval; DOAC, Direct Oral Anticoagulant; DVT, Deep Vein Thrombosis; IE, Index (VTE) Event; N, number of patients with available data; OR, Odds Ratio; PE, Pulmonary Embolism

Throughout the study observation period, 73.3% (355/484) of participants with available data were both adherent and persistent to their antithrombotic medication. The respective rates were 73.5% (136/185), 72.3% (175/242) and 77.2% (44/57) in the ‘DVT only’, ‘PE only’ and ‘DVT + PE’ subgroups, respectively; proportions were high in both DOAC- and non-DOAC treatment groups (Fig. 4A).

Univariable logistic regression analyses of factors associated with poor adherence and/or non-persistence to treatment are presented in Figure S2. Multivariable analysis showed that participants with diabetes mellitus had 51% lower odds of being poorly adherent and/or non-persistent to antithrombotic treatment than participants without diabetes (*p* = 0.038), and participants treated with ‘DOAC only’ had 43% lower odds of being poorly adherent and/or non-persistent to antithrombotic treatment than those receiving non-DOAC regimens or DOAC + any other anticoagulant medication (*p* = 0.027). Conversely, ever-smokers had 1.6-fold higher odds of being poorly adherent and/or non-persistent to antithrombotic treatment than never smokers (*p* = 0.025) (Fig. 4B).

## Discussion

The VICTORIA study, with 600 patients from leading vascular departments in Greece, provides insights into VTE patient profiles and treatment patterns. The results are generalizable, with a balanced distribution among the three specialties. The care setting was also balanced. In our study, all participants received anticoagulant therapy with a high proportion (27%) of parenteral only regimen compared to other studies (6–17%) in which however the percentage of hospitalized participants was not reported [20, 21]. A rate of 27% has also been reported in another Greek hospital-based cohort of persons with DVT [22] probably reflecting familiarity with the parenteral treatment. However, most participants received a combination of parenteral and oral anticoagulant and one third oral anticoagulant only patterns aligned with the European guidelines [3, 4]. A hypothesis could be that if oral AC only had been used, the length of hospital stay could have been shorter than the 5 days we reported.

Among patients treated with oral anticoagulant, the majority received DOACs (99%). This is in line with the reported proportion of DOAC-treated participants in other European/United States real-world studies, wherein the proportion ranges from 34%−92% [20–27] Apixaban has been the most frequently used DOAC recently [25–27].Similarly, apixaban was the most frequently administered DOAC in VICTORIA (65%) followed by rivaroxaban (36%). However, two thirds (67%) of apixaban-treated and nearly a third (29%) of rivaroxaban-treated participants started treatment with lower-than-recommended lead-in doses [28–31]. In most cases, an initial dose of parenteral heparin was administered. However, 20% of apixaban- and 3% of rivaroxaban-treated participants had not received any prior anticoagulant. It should be noted though that the majority of patients who have not received any loading dosage (either of DOAC or parenteral AC) were patients with ‘DVT only’. These are not new findings since in some real-world (RW) studies the proportion of participants not receiving the recommended lead-in doses ranges from 8%−22% [32–35] or even higher (50% for apixaban; 70% for rivaroxaban) [34–36], while 25–37% did not receive any lead-in anticoagulant [36, 37]. When asked about skipping the lead-in dose, VICTORIA physicians cited their clinical practice, high bleeding risk, presence of active cancer and previous treatment with parenteral AC Indeed, limited prior research indicates that factors like age, cardiac comorbidities, obesity, poor kidney function, history of major bleeding, concomitant antiplatelet medication, and cancer influence the decision to use reduced lead-in doses [33, 34, 37, 38]. Concerns about bleeding in high-risk individuals likely led to a conservative approach. However, the reasons for reducing or skipping the lead-in dose are not well-documented, warranting further research into this decision-making process.

Another interesting finding was the duration of treatment, which was identified as > 3 months in the majority of participants (91%) with an overall median of 6.0 months. Four out of ten participants (41%) received extended anticoagulant, while similar rates are also reported in the literature (41–57%) [20, 23]. Only 9% of the participants treated with DOACs (11% of apixaban-treated participants) received a reduced-dose schedule in the extended phase, as per guidelines [3, 5, 7] and labeling recommendations [18, 19]. Similarly, low rates of low dosage schemes have been reported in two other real world (RW) studies, but the rates were higher compared to our study (12–15% of participants, 24–28% in apixaban-treated participants) [23, 39]. This finding is crucial as it highlights the limited information on RW practices for selecting extended phase anticoagulant dosages. Further studies with longer follow-up and physician input on treatment decisions are needed to enhance our understanding of this clinical issue.

Among the factors that led to the decision to administer extended anticoagulant -based on our multivariable analysis- was the presence of persistent RFs, which could potentially increase the likelihood of VTE recurrence [3–5]. On the contrary, temporary minor RFs were less likely to drive the extended anticoagulant use, possibly balancing the benefit-to-risk of bleeding ratio. Additionally, extended anticoagulant was negatively associated with diabetes mellitus and COVID-19, which might be explained by an increased bleeding risk previously associated with diabetes [40–44], and a low risk of recurrent thrombosis in COVID-19 patients [45].

COVID-19 is considered a temporary RF by physicians [46, 47]. Notably, even though statistical analysis was not performed, the COVID-19 rate was much higher among PE ± DVT (27%) than DVT only (4%) in VICTORIA. Numerous studies have shown that VTE in persons with COVID-19 occurs more frequently as PE than DVT, supporting the proposed underlying pathophysiological mechanism of immunothrombosis in COVID-19 [46, 48–50].

With respect to the special population of participants with cancer, guidelines recommend LMWH or rivaroxaban/apixaban for the first 6 months, (depending on contraindications), while anticoagulant should be extended beyond 6 months or until cancer is cured [3, 7, 51]. Initial management in VICTORIA among participants with active cancer followed guidelines, as showcased by the higher rates of parenteral anticoagulant only compared with the overall population (59% versus 27%). Furthermore, active cancer was positively associated with extended anticoagulant in our study as demonstrated in the univariable analysis; though cancer could not be directly examined as an independent factor in the multivariable analysis since it also fell under the umbrella of persistent RFs, the latter emerging as an independent positive predictor. Altogether the above-described treatment practices depict how recommendations are translated by physicians into clinical practice, providing insights into the inconclusive literature data with respect to anticoagulant duration [13–16].

The rate of adherence/persistence throughout study participation for both DOAC and non-DOAC-treated subpopulations exceeded 72%, which appears high compared with the 6-month persistence rates of an earlier systematic review of observational studies (pooled meta-analysis: 62%) [52] and more recent studies in patients with acute VTE treated with oral anticoagulant (41%−89%) [25, 52–56] but we used a composite endpoint of both persistence and adherence, in contrast to other studies which addressed only persistence. Population differences (e.g. smoking habits, diabetes, and single-agent treatment) may also affect adherence/persistence as supported by our multivariable analyses results. Adjusted for age and temporary risk factors, patients treated with DOACs only, appear significantly less likely to be poorly adherent and/or non-persistent as also reported for cancer associated thrombosis patients [57]. Due to the differences in methodologies, VICTORIA findings for adherence cannot be appraised in relation to available literature [11, 56, 58–60]. Of note, contact by phone during follow-up may contribute to upward bias (false positive self-report of good adherence/persistence), since patients may feel the pressure to respond in a socially acceptable way (social desirability bias). However, this has not affected the reliability of VICTORIA findings given that the concordance between electronic prescription and self-report was >99%. The validity of our results is also reinforced by the fact that ≥90% of the participants attended the 3-month and 6-month VICTORIA visits.

Our study provides valuable insights. However, there are some limitations that should be considered: 1. As an observational study causality cannot be established. The associations found between treatment patterns and outcomes may be influenced by unmeasured confounding factors. 2. Selection bias: Although consecutive sampling was used to reduce selection bias, the study population may not fully represent all VTE patients, especially those managed outside of the clinics. Additionally, only patients who agreed to participate and gave consent were included, which could introduce consent bias. 3. Generalizability: The study was conducted in Greece, and the findings might not be fully generalizable to other populations or healthcare systems 4. Data Collection and Reporting: The reliance on patient self-reports for adherence and persistence introduces the risk of reporting bias. Patients may overestimate their adherence due to social desirability bias. Although electronic prescription records were used to verify adherence, discrepancies might still exist. 5. Variable Follow-up Periods: The study's follow-up was limited to 6 months. Longer-term outcomes and adherence beyond this period were not assessed, potentially missing changes in treatment patterns. 6. Missing Data: with respect to key study objectives was low; none exceeded 1%, apart from the ARMS score at the 6-month visit, leading to an overall missing data rate for adherence/persistence outcomes of 10%, 14%, 8%, and 3% of the overall, ‘DVT only,’ ‘PE only’, and ‘DVT + PE’ patients, respectively. In any observational study, missing data can be a significant issue. Although the study attempted to minimize this, some variables may still have missing values, which could affect the robustness of the findings, especially in the multivariable analyses. 7. Physician Practices: The study highlights that many patients received lower-than-recommended lead-in doses of DOACs. However, the rationale behind physicians'decisions was not thoroughly investigated, leaving a gap in understanding the clinical decision-making process. 8. Impact of COVID-19: The study was conducted during the COVID-19 pandemic, which may have influenced both the incidence of VTE and the management practices. The findings might be different in a non-pandemic setting. 9. Sample Size for Subgroup Analyses: Some subgroups (e.g., those on extended reduced-dose DOACs) had relatively small sample sizes, which may limit the statistical power and reliability of the findings for these groups.

Despite these limitations, the VICTORIA study provides important real-world data on VTE treatment patterns, contributing to a better understanding of clinical practices and patient outcomes in this population.

Although, at the time the study was conducted all DOACs were available in Europe [28, 29] and the US [30, 31], comparisons should be cautiously interpreted in the context of different study designs. Also, eligibility criteria (e.g. active cancer excluded in some studies [20, 25, 27]), lack of detailed treatment sequences [20–22] and healthcare settings—inpatient cases may represent more severe VTE – may comprise additional confounding factors.

It is noteworthy to mention that the rate of extended anticoagulant which was calculated based on anticoagulant therapy status (ongoing/discontinued at the 6-month study visit), may have been overestimated considering the ± 1 month visit time window applied. Specifically, 25% of participants completed the 6-month visit in < 6 months from the IE. The magnitude of this bias is unclear but should be taken into consideration when interpreting the respective study findings.

The actual sample size (*N* = 600) corresponds to the expected one, thus enhancing the precision of the estimations, especially if we also take into consideration the high attendance rates of both follow-up visits (≥ 90%). Additionally, the precision of qualitative outcomes for the ‘DVT only,’ and ‘PE only’ subpopulations is deemed adequate given that their size ensured a margin of error below 7%. However, outcomes in the ‘DVT + PE’ subgroup should be interpreted with caution considering the relatively small sample size (*N* = 61).

## Conclusion

In summary, the VICTORIA study provides RW data on the management of acute VTE in Greece, focusing on anticoagulant type, dosage, and duration. The findings indicate a mixed parenteral/oral treatment approach for the acute phase, with DOACs being the most used oral therapy. However, standard and extended dosage schemes did not fully align with current guidelines. Extended anticoagulant use is high and depends on the presence of persistent risk factors, with high adherence and persistence rates across treatment regimens.

## Supplementary Information

Supplementary Material 1.

Supplementary Material 2.

## Data Availability

No datasets were generated or analysed during the current study.
