# Supplementary figures and images for: Α 6-month, multicenter, observational study investigating the treatment of venous thromboembolism in Greece (VICTORIA study)
Source: Thromb J. 2025 Jun 23;23:71. doi: 10.1186/s12959-025-00749-1 (PMC12183886; doi:10.1186/s12959-025-00749-1)

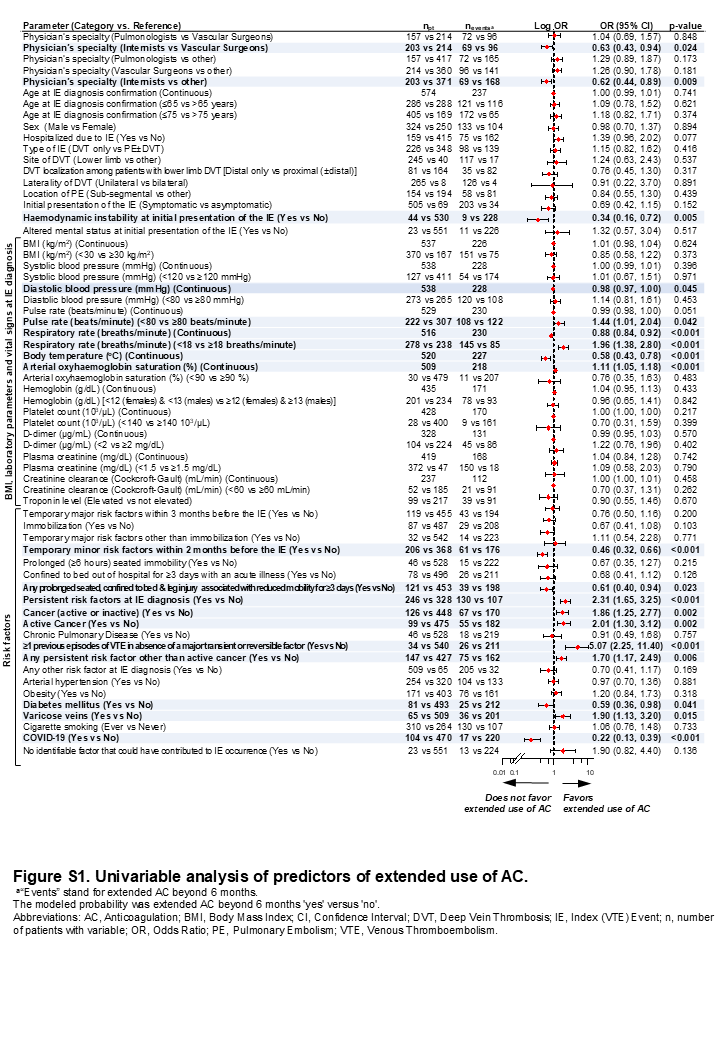

Supplement: Supplementary file 1 — Supplementary Material 1. [file 12959_2025_749_MOESM1_ESM.tif]

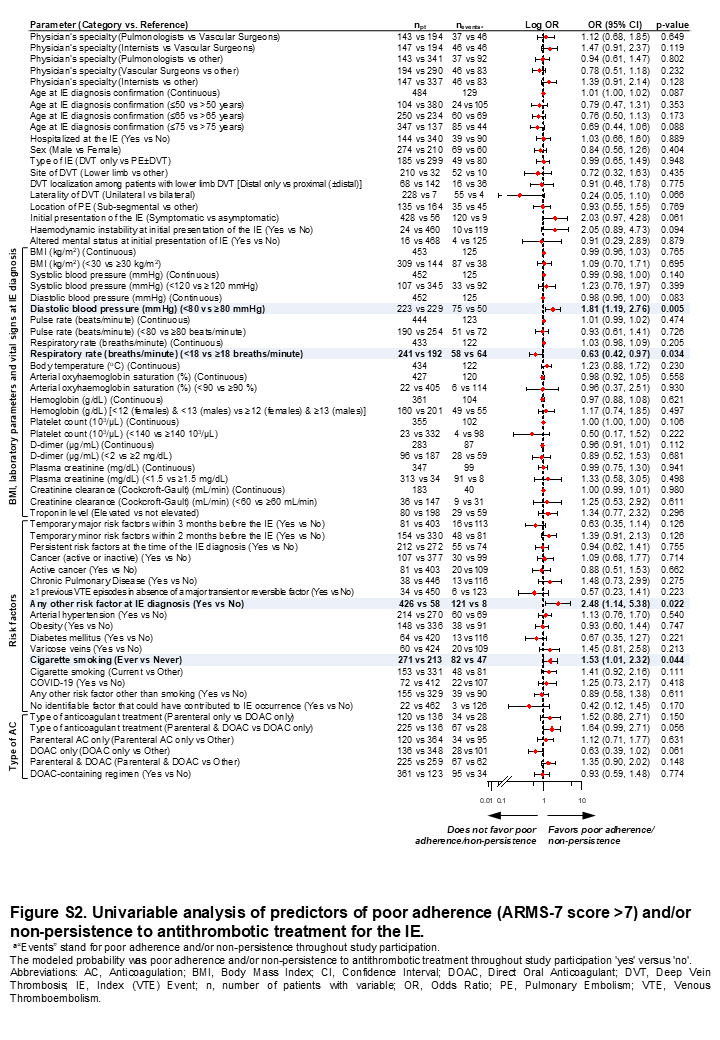

Supplement: Supplementary file 2 — Supplementary Material 2. [file 12959_2025_749_MOESM2_ESM.tif]
